# Supplementary material for: Mumefural Ameliorates Cognitive Impairment in Chronic Cerebral Hypoperfusion via Regulating the Septohippocampal Cholinergic System and Neuroinflammation
Source: Nutrients. 2019 Nov 13;11(11):2755. doi: 10.3390/nu11112755 (PMC6893811; doi:10.3390/nu11112755)
Supplement: Supplementary file 1 [file nutrients-11-02755-s001.pdf]

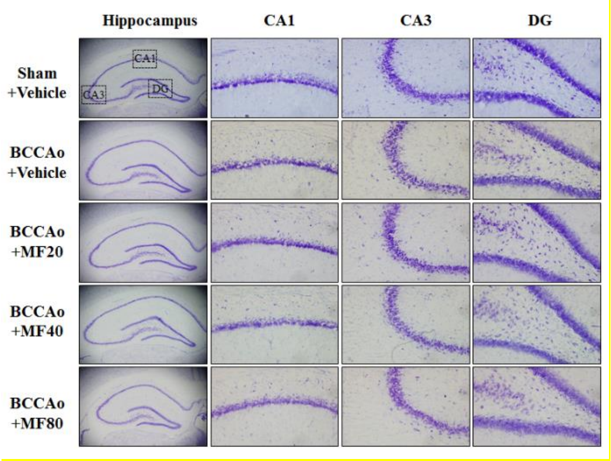

Figure S1: MF does not affect neuron density in the sub-regions of the hippocampus in BCCAo rats. Representative images of Nissl staining in the hippocampus under 40 × and 100 × magnifications.
